# Ultrasonographically detected hepatosteatosis independently predicts the presence and severity of coronary artery disease

**DOI:** 10.4314/ahs.v22i2.31

**Published:** 2022-06

**Authors:** Aykut Yılmaz, Fevzi Yılmaz, İnan Beydilli, Bedriye Müge Sönmez, Murat Duyan, Metin Özdemir, Seval Komut, Yüksel Aksoy

**Affiliations:** 1 Training and Research Hospital, Department of Cardiology, Siirt /Turkey; 2 Antalya Education and Research Hospital; Department of Emergency Medicine, Antalya /Turkey; 3 Numune Education and Research Hospital; Department of Emergency Medicine, Ankara /Turkey; 4 İstanbul EsenyurtNecmiKadıoğlu State Hospital, Department of Emergency Medicine, İstanbul /Turkey; 5 Hitit University, ErolOlçok Training and Research Hospital, Department of Emergency Medicine, Corum, Turkey; 6 Trakya Unıversıty Faculty of Medicine, Department of Cardiology, Edirne /Turkey

**Keywords:** Nonalcoholic fatty liver disease, gensini score, obesity

## Abstract

**Background:**

Nonalcoholic fatty liver disease (NAFLD) has shown to be associated with coronary artery disease (CAD)

**Objectives:**

The aim of our study was to evaluate the association between the presence and severity of CAD and NAFLD.

**Methods:**

The study group consisted of 153 patients who underwent coronary angiographies. Patients were categorized into CAD and non-CAD groups. CAD severity was determined by the number of CAD-involved arteries and the vessel score multiplied by Gensini score, the latter judging CAD severity. Fatty liver was diagnosed by abdominal ultrasonography (USG), with the patients being categorized by the degree of hepatosteatosis, as Grade 0, Grade 1, and Grade 2–3.

**Results:**

Among the whole study population, 47.1% of patients (n=72) were female and 52.9% of patients (n=81) were male. Forty-three patients had normal coronary arteries; 27 patients had non-critical CAD and side branch disease; and 83 patients had clinically significant CAD (stenosis>50%). The rate of CAD and Gensini score were significantly different between Grade 0, 1 and 2–3 hepatosteatosis groups (p<0.05). Patients with CAD had a significantly higher AST level than those without (p< 0.05).

**Conclusions:**

Ultrasonographically detected hepatosteatosis independently predicts the presence and severity of CAD.

## Introduction

Coronary artery disease is one of the commonest causes of mortality and morbidity in developed societies. Although major risk factors of the disease are known, classical risk factors alone may remain incapable of explaining the cause of CAD seen in some patients. It has been reported that about half of patients suffering an acute coronary syndrome (ACS) do not carry classical cardiovascular risk factors.^1^ Nowadays, novel risk factors apart from classical risk factors for CAD are under scrutiny.

Nonalcoholic fatty liver disease (NAFLD) means hepatic steatosis due to fat accumulation without seconder causes as heavy alcohol consumption. NAFLD is classified as nonalcoholic fatty liver (NAFL) and nonalcoholicsteatohepatitis (NASH). The difference is the presence of hepatic inflammation in NASH where the steatosis is very similar to alcoholic steatohepatitis histologically.^2^ Other terms that have been used to describe NASH include pseudoalcoholic hepatitis, alcohol-like hepatitis, fatty liver hepatitis, steatonecrosis, and diabetic hepatitis.

NAFLD is a clinical and pathological condition associated with abdominal obesity, type 2 diabetes mellitus (DM), hypertension (HT), and dyslipidemia. While NAFLD affects 14–23 % of the general population, with the figure rising up to 70–90 % among obese and type 2 diabetic persons.^3^,^4^ Whereas NAFLD and CAD share many risk factors in common, only a few trials have studied the direct relation between the two.^5^,^6^

NAFLD is usually accompanied with metabolic syndrome (MetS) (obesity, systemic hypertension, dyslipidemia, Insulin resistance or overt diabetes) as mentioned in the study consists of 304 patients with NAFLD but without overt diabetes.^7^ 120 (74%) of 163 liver biopsies were histologically revealed as NASH. 53 percent of the remained unbiopsied, 67 percent of the patients whom diagnosed as NAFL and 88 percent of those with NASH on biopsy were diagnosed as MS. Statistical analyses with adjusted age, sex, and body mass index (BMI), MetS was associated with an increased risk of severe fibrosis (odds ratio [OR] 3.5, 95% confidence interval [CI] 1.1–11.2), hence NAFLD and cardiovascular disease (CVD) may be independently associated as mentioned in NHANES data (odds ratio 1.23; 95% CI 1.04–1.44).^8^

Hence, this study primarily aimed to investigate if there was any association between NAFLD, CAD presence and severity, and obesity. It also aimed to evaluate relationship between CAD and NAFLD and serum levels of AST, ALT, and GGT.

## Methods

This study was approved by the local hospital's ethics committee and enrolled patients who underwent coronary angiography at Trakya University Faculty of Medicine Hospital for various indications like ACS, chest pain, and/or positive exercise stress test or abnormal nuclear imaging.

### Inclusion criteria included the following

Patients should have undergone coronary angiography, being free of any history of alcohol intake or known liver disease, congestive heart failure, corpulmonale, cancer, and acute or chronic infectious disorders. We excluded patients without coronary angiography, with an alcohol intake and those who reported a history of known liver disease, pregnancy, congestive heart failure, severe pulmonary disease, corpulmonale, chronic renal failure, cancer, active infection, and hepatosteatosis induced by drugs such as steroids. Those who did not a hepatic USG or adequate ultrasonic images of the liver, those with a positive HAS test (hepatitis B and C, HIV, and syphilis tests), and those with deficiencies in the identified biochemical studies were left out of the study. The limit level of alcohol consumption was calculated as 30 grams of alcohol per day for men and 20 grams for women.

Clinical and demographic characteristics of enrolled patients were identified. Age, sex, HT, DM, weight, height, Body mass index (BMI), family history of CAD, smoking status, lipid profile, urea and creatinine levels, medications used; anti-lipid medications and other drugs (such as nitrates, beta blockers, calcium channel blockers, angiotensin converting enzyme inhibitors or angiotensin II receptor blockers, antiaggregant and antidiabetic medications) were screened and recorded. Patients with a BMI ≥30 kg/m2 were considered as obese.^6^

### Laboratory Tests

All laboratory tests were performed at Trakya University Faculty of Medicine Central Laboratory, using Siemens Advia 1800 autoanalyzer (Siemens Healthcare Diagnostics, Tarrytown NY) device and the kits of the same brand pertaining to the same device. Per blood collection protocol, all blood samples for studying routine biochemistry and total cholesterol, low-density lipoprotein (LDL) and high-density lipoprotein (HDL) cholesterols, triglycerides, AST, ALT, and GGT levels were taken within 24 hours of hospital admission. NCEP/ATP III guidelines were taken reference when diagnosing dyslipidemia and MetS.^9^

### Ultrasonographic examination

Abdominal ultrasonography was performed in patients afer overnight fasting, in supine position or in the left decubital position to achieve the best possible visualization of the liver. Te USG probe was lubricated with gel to avoid artifacts from air and dry skin surfaces. All patients' hepatobiliary USG studies were carried out using Siemens Acuson X300 and SaoteMylab 60 ultrasonography devices. Hepatobiliary USG was performed shortly before the patient was discharged and after a fasting period of 12 hours, by a radiologist who did not have information about the patient.

In comparison with kidney cortex in grey scale, fatty liver is brighter. Proportionally, the more fatty liver the less visualization of the deeper parts of liver. In comparison with right kidney cortex increased echogenicity of liver parenchyma means fatty liver. Visibility and sharpness of diaphragm and hepatic veins can interface. Grading of the fatty liver is made according to these parameters: Grade 0: no steatozis (liver and renal cortex have the same echogenicity; Grade 1: mild steatozis (liver cortex is slightly brighter, diaphragm visualized clearly and the contors of hepatic veins have sharp interface) Grade 2: moderate steatozis; (at the deeper parts of the live US beam is attenuated, the contors of the diaphragm and hepatic veins are blunted); Grade 3: severe steatozis (very bright liver parenchyma, severe attenuation, contors are not seen).^9^

In order to minimize errors originated from the subjective criteria used for the ultrasonographic evaluation of hepatosteatosis, NAFLD was further graded into Grade 1–2 (mild-no hepatosteatosis) and Grade 2–3 (moderate-severe hepatosteatosis) groups.

### Coronary angiography

Selective coronary angiographic evaluations of the right and left coronary systems were carried out with the Judkins technique and Philips Integris H 3000 (Eindhoven, The Netherlands) and Siemens Artis Zee (Germany) angiography devices. Two cardiologists experienced in performing and interpreting angiographic studies but who were blinded to the study design evaluated the presence and severity of CAD. On coronary angiograms coronary stenoses of 50% or more in at least one epicardial coronary artery or its major branches was considered CAD.^10^ CAD extent and severity were evaluated with Gensini score.^11^ The latter was calculated by taking into account the Grade and regional importance of stenoses in epicardial arteries. Luminal stenoses of 25%, 50%, 75%, 90%, 99%, and 100% were assigned stenosis scores of 1, 2, 4, 8, 16, 32, respectively.

Each narrowed coronary artery region was assigned a significance coefficient depending on the functional importance of the myocardial region supplied by that vessel. Five points were assigned to left major coronary artery (LAD) segment; 2.5 for proximal LAD segment; 1.5 for medial LAD segment; 1.5 for distal LAD segment; 1 for diagonal LAD segment; 2.5 for proximal circumflex artery segment; 1 for obtuse marginal and posterolateral branch segments; 1.5 fr right proximal coronary segment; 1 for posterior descending artery segment; and 0.5 for other segments. For all stenoses, stenosis scores were multiplied by the functional significance coefficients and the results were summed up to obtain Gensini score.

### Study Groups

Two main groups of patients based on the presence of CAD were formed, namely the CAD (83 patients) and non-CAD (70 patients) groups, which were further divided by the number of affected vessel [one-, two-, or three-vessel disease (1VD, 2VD or 3VD)]. Additionally, other categorizations were also done by the existence of obesity and existence and severity of hepatosteatosis. After calculation of Gensini score, its median value of 36 points was determined as the cut-off value for grouping patients on the basis of atherosclerosis severity; hence, ≤36 points indicated absent or mild CAD (mean: 9.7+10.8) and >36 points indicated medium-to-severe CAD (mean: 87.0+36.5).

### Statistical evaluation

Statistical analyses of the study data were performed using S0064 Minitab Release 13 (Licence No: wcp1331.00197) statistical software operated at the data processing center of the Trakya University Faculty of Medicine Deaconship. Quantitative data were presented as mean+standard deviation and qualitative data as frequency and percentage. Data distribution was tested using Kolmogorov-Smirnov test. Quantitative variables were analyzed using independent samples t test and Mann Whitney-U test. Qualitative variables were analyzed using Chi-square test. Correlation tests were performed using Spearman's correlation test. p<0.05 was considered statistically significant.

## Results

This study included 153 patients who underwent both hepatobiliary USG and coronary angiography. The clinical and demographic properties, biochemical, ultrasonographic, and angiographic data of the study population were compared by the presence of CAD. Of all patients, 47.1% (n=72) were female and 52.9% (n=81) were male. Forty-three patients had normal coronary arteries; 27 patients had non-critical CAD and side branch disease; and 83 patients had clinically significant CAD (>50% stenosis). Of the whole study population, 75.8% (n=116) had HT; 28.1% (n=43) had DM; and 32.5% had history of or ongoing MI. Thirty-one percent (n=48) of the patients had Grade 0 hepatosteatosis; 42.5% (n=65) Grade 1 hepatosteatosis ; %48.2% (n=40) had Grade 2–3 hepatosteatosis (Table 1).

**Table 1:** Clinical, laboratory and angiographic findings in patients undergoing coronary angiography

		Coronary Artery Disease		

		Absent (n=70)	Present (n=83)	P
			
Parameters		Mean±s.d. / n-%	Mean±s.d. / n-%	
Age, years		59,6±10,7	62,3±9,4	0,098

Gender, n(%)	Women	43 (61,4%)	29 (34,9%)	**0,001**
	Men	27 (38,6%)	54 (65,1%)	

Body mass index, kg/m^2^		29,4±4,4	28,9±4,1	0,489

Smoking, n(%)		30 (42,9%)	49 (59,0%)	**0,046**

Triglycerides, mg/dL		138,4±62,7	155,5±74,2	0,129
Total cholesterol, mg/dL		181,9±38,9	181,0±36,4	0,882
HDL cholesterol, mg/d		47,1±11,7	40,3±11,1	**0,000**
LDL cholesterol, mg/dL		120,0±36,8	126,3±36,5	0,296
AST, U/L		23,3±12,4	26,7±11,6	**0,023**
GGT, U/L		30,3±19,2	37,3±38,7	0,116

Family history, n(%)		12 (17,1%)	49 (59,0%)	**0,000**
Hypertension, n(%)		49 (70,0%)	67 (80,7%)	0,123
Diabetes mellitus, n(%)		17(24,3%)	26 (31,3%)	0,335
Prior MI, n(%)		0(0,0%)	27 (32,5%)	**0,000**
Dyslipidemia, n(%)		25(35,7%)	15 (18,1%)	**0,013**
Anti-lipid drugs, n(%)		22(31,4%)	36 (43,4%)	0,129
Other drugs, n(%)		48(68,6%)	63 (75,9%)	0,311

Hepatosteatosis	Grade 0	28 (40,0%)	20(24,1%)	**0,000**
	Grade I	42 (60,0%)	23(27,7%)	
	Grade II–III	0 (0,0%)	40(48,2%)	

**DM:** Diabetes mellitus, **HT:** Hypertension, **MI:** Myocardial infarction, **HDL:** High density lipoprotein, **LDL:** low density lipoprotein, **AST:** Aspartate aminotransferase, **GGT:** Gama-glutamyl transferase, Obesity- body mass index >30 kg/m^2^. Other drugs are given in the text.

**Figure 1:**
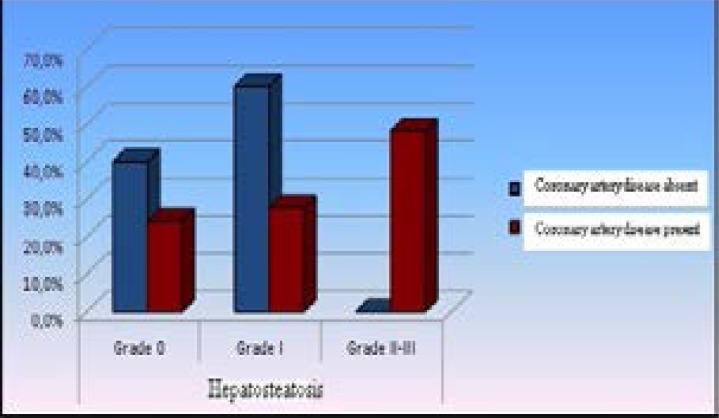
Distribution of CAD prevalence by hepatosteatosis grade

The ratio of males was higher in patients with CAD than those without (p<0.05). Patients with and without CAD showed no significant differences with respect to age and BMI (p>0.05). Patients with CAD had a significantly higher smoking rate than those without (p<0.05). Patients with and without CAD showed no significant difference with respect to triglyceride, total cholesterol, LDL, and GGT levels (p>0.05). AST level was significantly higher in patients with CAD than those without (p<0.05). Patients with CAD had a significantly lower HDL level than patients free of CAD (p<0.05). HT, DM, antilipidemic medication or use of other drugs were not significantly different between the two groups (p>0.05). Patients with CAD had a significantly higher rate of family history for CAD than those without (p<0.05). Dyslipidemia was significantly more common among patients with CAD than those without (p<0.05). The CAD group had a significantly greater rate of Grade II-III hepatosteatosis than the non-CAD group (p < 0.05) (Table 1, Figure 1).

Table 2 shows the comparison of clinical, biochemical, ultrasonographic, and angiographic findings by hepatosteatosis grade. Age and smoking rate did not show significant difference by hepatosteatosis grade (p>0.05). Patients with Grade II-III hepatosteatosis had a greater male gender ratio than patients with Grade I hepatosteatosis (p< 0.05). Sex distribution of patients with Grade 0 hepatosteatosis was similar to that of patients with Grade I or Grade II-III hepatosteatosis (p>0.05). Patients with Grade 0 hepatosteatosis had a significantly lower BMI than those with Grade I and Grade II-III hepatosteatosis (p< 0,05). Patients with Grade I and Grade II-III hepatosteatosis had similar BMI (p > 0.05).

**Table 2:** Clinical, laboratory and angiographic findings according to fatty liver grades

		Hepatosteatosis						

Parameters		Grade 0 (n=48)		Grade I (n=65)		Grade II–III (n=40)		p
		
		Mean±s.d.	/ n-%	Mean±s.d.	/ n-%	Mean±s.d.	/ n-%	
Age		59,9	± 11,9	61,0	± 9,2	62,5	± 9,2	0,499

Sex	Female	21	43,8%	39	60,0% *	12	30,0%	**0,010**
	Male	27	56,3%	26	40,0%	28	70,0%	

BMI		27,2	± 3,4*‡	30,0	± 4,4	30,2	± 3,9	**0,000**

Smoking		25	52,1%	29	44,6%	25	62,5%	0,204

Triglyceride		127,4	± 50,6*	149,4	± 78,9	169,3	± 67,2	0,017
Total Cholesterol		184,2	± 41,6	179,6	± 40,6	180,9	± 25,4	0,813
HDL		45,9	± 12,8*	45,2	± 11,5*	37,5	± 9,1	**0,001**
LDL		125,4	± 39,6	119,1	± 38,7	128,1	± 29,0	0,437
AST		27,0	± 15,9*	21,9	± 6,8	28,3	± 12,3	0,024
GGT		31,6	± 18,4	30,6	± 22,4	42,8	± 50,0	0,144

HT		28	58,3%*‡	54	83,1%	34	85,0%	**0,003**
DM		5	10,4%*‡	24	36,9%	14	35,0%	**0,004**
MI		6	12,5%*	7	10,8%*	14	35,0%	**0,004**
Family history		7	14,6%*‡	23	35,4%*	31	77,5%	**0,000**
Dyslipidemia		29	60,4%*	47	72,3%*	37	92,5%	**0,003**
Antilipidemic drug use		13	27,1%	26	40,0%	19	47,5%	0,130
Use of other drugs		26	54,2%*‡	55	84,6%	30	75,0%	**0,001**

Gensini Score		9,8	± 11,9*	12,7	± 18,7*	56,7	± 26,4	**0,000**
Gensini Score	≤ 36	47	97,9%*	60	92,3%*	4	10,0%	**0,000**
	36 <	1	2,1%	5	7,7%	36	90,0%	

Kruskal-wallis (Mann-whitney u test) / Chi-square test / * p < 0.05 vs Grade II-III

Chi-square test / * p < 0.05 vs Grade II–III / ‡ p < 0.05 vs Grade I

**BMI:** Body mass index. **HDL:** High density lipoprotein, **LDL:** low density lipoprotein, **AST:** Aspartate aminotransferase, **GGT:** Gama-glutamyl transferase. **DM:** Diabetes mellitus, **HT:** Hypertension, **MI:** Myocardial infarction, Other drugs are given in the text.

Total cholesterol, LDL, or GGT levels did not show significant difference by hepatosteatosis grade (p>0.05). Triglyceride levels of patients with Grade II-III hepatosteatosis were significantly higher than those with Grade 0 hepatosteatosis (p<0.05). HDL level was significantly lower in the group with Grade II-III hepatosteatosis than the groups with Grade I and Grade 0 hepatosteatosis (p<0.05). Patients with Grade II-III hepatosteatosis had a significantly higher AST level than patients with Grade 0 hepatosteatosis (p<0.05). Patients with Grade 0 hepatosteatosis had significantly lower rates of HT and DM than patients with Grade I and Grade II-III hepatosteatosis (p<0.05). Grade I and Grade II-III hepatosteatosis groups showed no significant difference with respect to the rates of HT and DM (p>0.05). Grade II-III hepatosteatosis group had a significantly higher dyslipidemia and MI rate than Grade 0 and Grade I hepatosteatosis groups (p<0.05). Patients with Grade 0 hepatosteatosis had significantly lower rates of familhistory for CAD than those with Grade I and Grade II-III hepatosteatosis (p<0.05). Family history for CAD was significantly lower in patients with Grade I hepatosteatosis than patients with Grade II-III hepatosteatosis (p<0.05). The rate of antilipidemic drug use did not show significant difference by hepatosteatosis grade (p>0.05).

**Figure 2:**
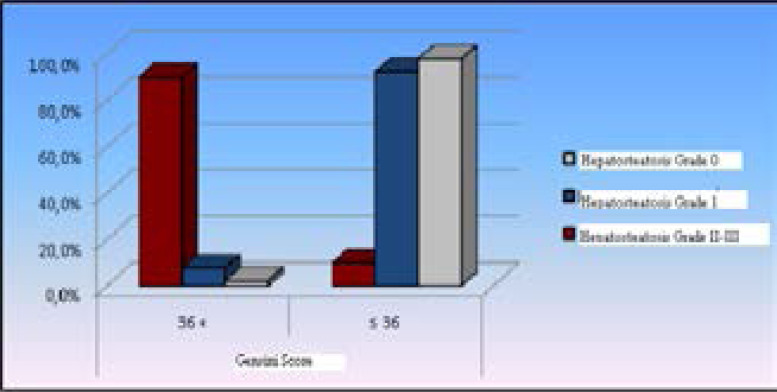
Distribution of hepatosteatosis prevalence by grouped Gensini score

**Figure 3:**
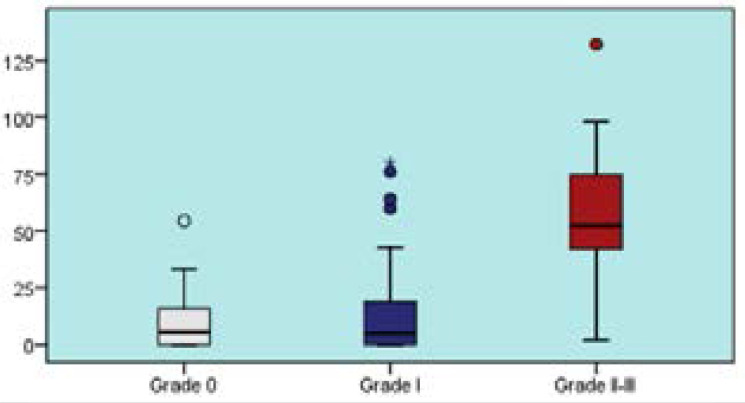
Relationship between hepatosteatosis grade and Gensini score

Patients with Grade II-III hepatosteatosis had a significantly higher mean Gensini score and a significantly higher rate of patients having a Gensini score >36 than patients with Grade 0 and Grade I hepatosteatosis (p<0.05), (Table 2, Figure 2, 3).

**Figure 4:**
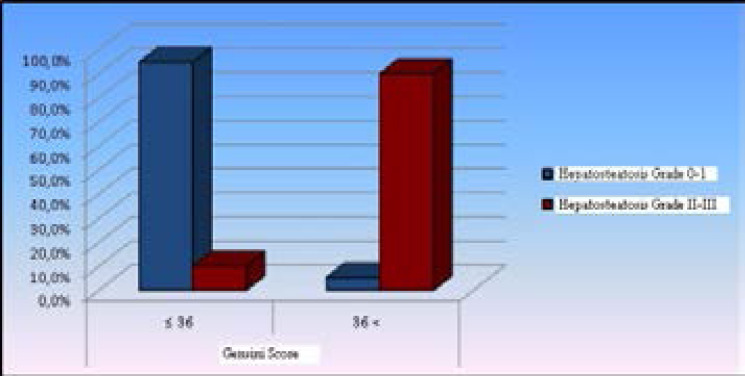
Distribution of the rates of Grade 0-I and Grade II-III hepatosteatosis by grouped Gensini score

As previously stated in the material-method section, patients that were grouped as Grade 0-I hepatosteatosis and Grade II-III hepatosteatosis were compared with respect to both numerical Gensini score and by the groups with a Gensini score ≤ 36 vs. a Gensini score >36. Patients with Grade II-III hepatosteatosis had a significantly higher mean Gensini score and the ratio of patients with a Gensini score >36 thanthose with Grade 0-I hepatosteatosis (p< 0.05), (Table 3, Figure 4).

**Table 3:** Comparison of patients categorized as Grade 0–I and Grade II–III hepatosteatosis for Gensini score

		Hepatosteatosis				

		Grade 0–I (n=103)		Grade II–III (n=40)		p
			
		n	%	n	%	
Gensini Score		11,4	± 16,2	56,7	± 26,4	**0,000**
Gensini Score	≤ 36	107	94,7%	4	10,0%	**0,000**
	36 <	6	5,3%	36	90,0%	

Mann-Whitney U test / Chi-square test

A comparison of the groups with Gensini score ≤ 36 and >36 revealed that total cholesterol, LDL, GGT levels were not significantly different (p>0.05). The group with Gensini score >36 had significantly higher triglyceride and AST levels compared to the group with Gensini score ≤ 36 (p<0.05). HDL level was significantly lower in patients with Gensini score >36 than those with Gensini score ≤ 36 (p<0.05) (Table 4).

**Table 4:** Comparison of biochemical parameters by grouped Gensini score

	Gensini Score		

	≤ 36 (n=101)	36 < (n=42)	p
		
	Mean±s.d.	Mean ±s.d.	
Triglyceride	140,1 ± 69,0	169,3 ± 67,2	**0,022**
Total Cholesterol	181,5 ± 40,9	180,9 ± 25,4	0,920
HDL	45,5 ± 12,0	37,5 ± 9,1	**0,000**
LDL	121,8 ± 39,0	128,1 ± 29,0	0,351
AST	24,0 ± 11,8	28,3 ± 12,3	**0,048**
GGT	31,0 ± 20,8	42,8 ± 50,0	0,359

Independent samples t test / Mann-Whitney U test.

**HDL:** High density lipoprotein, **LDL:** low density lipoprotein, **AST:** Aspartate aminotransferase, **GGT:** Gama-glutamyl transferase

The correlation analyses showed that Gensini score had no significant correlation to age, BMI, total cholesterol, LDL, and GGT levels (p> 0.05). While there was a positive correlation between Gensini score and the levels of triglycerides, AST (p< 0.05), a negative correlation existed between Gensini score and HDL level (p < 0.05) (Table 5).

**Table 5:** Comparison of Gensini score with clinical and biochemical parameters

		Age	BMI	Triglyceride	Total Cholesterol	HDL	LDL	AST	GGT
**Gensini Score**	r	0,151	-0,035	0,179	0,006	-0,361	0,116	0,217	0,159
	p	0,062	**0,667**	0,027	0,945	**0,000**	0,152	**0,007**	0,051

Spearman's Correlation test.

**BMI:** Body mass index, **HDL:** High density lipoprotein, **LDL:** low density lipoprotein, **AST:** Aspartate aminotransferase, **GGT:** Gama-glutamyl transferase

## Discussion

In the light of the present findings and scientific bases in our study primarily; between the prevalence and severity of coronary atherosclerosis and NAFLD; secondarily, it was aimed to investigate whether there is a significant relationship between AST and GGT levels. According to the results of our study, the rate of patients with grade 2 and grade 3 hepatosteatosis was significantly higher in the CAD group than the non-CAD group. When the prevalence and severity of atherosclerosis was evaluated using the Gensini score, the Gensini score was found to be significantly higher among the groups as the hepatosteatosis grade increased. When the patients are grouped as grade 0–1 and grade 2–3 according to hepatostatosis and ≤ 36 (no coronary atherosclerosis or mild) and > 36 (moderate and severe coronary atherosclerosis) according to the Gensini score, the ratio of patients with Gensini score mean and Gensini score > 36 in the group with hepateastosis grade 2–3 was significantly higher than the group with hepasteastosis grade 0–1 (p <0.05). An analysis of our secondary objective, i.e the relationship of NAFLD and CAD with liver enzymes, reveals that among patients with CAD, as compared to those without, AST level was significantly higher while GGT levels were similar. When correlated with Gensini score, ALT level showed a significant correlation but GGT level did not, although a trend for statistical significance was observed (p=0.051).

We found significantly higher rates of smoking, male gender, previous MI, family history of CAD, and dyslipidemia in patients with CAD (>50% stenosis); HDL level was significantly lower. A comparison using Gensini score still showed a significant correlation with triglyceride and HDL levels. As statistically significant parameters are known risk factors for CAD, this was consistent with our current knowledge. We found no significant relationship between CAD and HT, DM, BMI, other serum lipid parameters, antilipidemic drugs, and the rate of use of other medications (nitrates, beta blockers, Ca channel blockers, ACE-inhibitors, AT- 2 blockers, antiaggregants and antidiabetics).

A strong correlation has been shown between NAFLD and coronary risk factors such as endothelial dysfunction and subclinical atherosclerosis markers such as increased carotid intima media thickness.^12^ Targher et al.^13^ reported that patients with biopsy-proven NAFLD had a significantly greater carotid IMT. Chambless et al.^14^ followed 7865 women and 6349 men for 6–9 years to investigate the relationship between carotid IMT and stroke risk in the ARIC (Atherosclerosis Risk in Communities) study. They reported a significantly greater carotid IMT in patients with NAFLD than the healthy group matched for age, sex, and BMI. Both the increase in intima-media thickness and endothelial dysfunction are accepted as indicators of early atherosclerosis. Due to its relationship with these subclinical atherosclerosis markers, it has been reported that NAFLD may also be a risk factor for CAD. A study scrutinizing the relationship between the extent of hepatosteatosis and the extent of coronary atherosclerosis assessed by Gensini score among patients diagnosed with NAFLD similarly showed a significant positive relationship between ultrasonographically determined NAFLD, its severity and Gensini score. 15

Treeprasertsuk et al.^16^ in a review dated 2010 on studies on NAFLD and CAD, reported that patients with NAFLD, as compared to those without, had suffered a greater incidence of cardiovascular events but the relationship between histological progression of NAFLD and CAD events was not linear. The authors also stressed the importance of the need for conducting further studies to show a direct correlation between NAFLD and CAD. In another study, Assy et al.^17^ studied the relationship between NAFLD diagnosed by Computerized Tomography (CT) and coronary atherosclerosis diagnosed by CT Angiography and insulin resistance, C-reactive protein, and lipid profile. They found a significantly greater rate of calcified and non-calcified plaque burden determined by CT coronary angiography, non-obstructive CAD, insulin resistance, and triglyceride level among patients with NAFLD, patients with NAFLD were found to be at a greater risk of coronary atherosclerosis.

A few available studies in the literature have supported our direct findings that indicate a positive significant correlation between the extent of coronary atherosclerosis determined by Gensini score and NAFLD.^13^,^18^ Targher et al.^13^ reported that patients with NAFLD had a higher risk of fatal and / or non-fatal CVD events than those without NAFLD. Patients with more ‘severe’ NAFLD were also more likely to develop fatal and non-fatal CVD events. Rinella Reported.^19^ A systematic review and also reported that the association between nonalcoholic steatohepatitis and CVD is clear, though causality remains to be proven in well-controlled prospective studies. Both systemic rewievs support the positive significant relationship between CAD and NAFLD that we found in our study.

In determining the risk for CAD, visceral obesity is among the major risk factors as well as many other risk factors. At the same time, the increase in visceral adipose tissue has been found to be associated with accelerated atherosclerosis.^20^ Therefore, NAFLD is also called the hepatic reflection of the MetS.^18^,^21^ More than 90% of people with NAFLD have at least one component of the MetS and 1/3 of them have MetS. In addition, obesity has been shown to increase morbidity and mortality, both as a component of the MetS and with CVD; It has been stated that BMI is a risk factor for unstable angina and MI in patients with angiographically proven CAD^21^,^22^. Ballestri at al.^23^ published a metaanalyses and systematic review of the literatüre they gauged the risk of developing Type 2 DM and MetS in patients with NAFLD, reported that NAFLD was significantly associated with incident Type 2 DM and with incident MetS. Lonardo et al.^24^ published systematic review of the literature emphasize that the presence of NAFLD is intimately linked with the MetS; NAFLD is a strong determinant for the future development of the MetS. Ballestri at al.^25^ researched that relationship of serum Fetuin-A leves with coronary atherosclerotic burden and NAFLD in patients undergoing elective coronary angiography; reported that BMI, waist circumference, TGs levels, fasting glucose, HOMA, spleen area, and SAT thickness, as well as the prevalence of metabolic derangements (hyperlipidemia, DM, and MetS) were significantly higher in NAFLD patients. In our study, besides the positive significant relationship between gensini score and NAFLD, MetS parameters such as HT, dyslipidemia, DM patients rate and BMI were found to be significantly higher in the group with grade 2–3 hepatosteatosis.

Lonardo et al.^26^ published another systematic review of the literatüre and reported that; NAFLD may be both a consequence and a cause of MetS and its individual components, and that the link between NAFLD/ NASH and HTN, T2DM and atherosclerosis / CVD is more complex than previously believed. A growing body of clinical and experimental evidence suggests that NAFLD may precede and/or promote the development of HTN, T2DM and CVD. They reported that the risk of developing these cardiometabolic diseases parallels the underlying severity of NAFLD. These findings supported the positive significant relationship we found between CAD and gensini score and existing MetS risk factors in our study. On the other hand there are studies in the literature to determine whether NAFLD has a genetic predisposition. Yan at al.^27^ researched that investigated the relationship of TCF7L2rs7903146 gene polymorphism with the risk of NAFLD, CAD, and NAFLD + CAD. Although previous studies have suggested that TCF7L2 rs7903146 was related to the risk of developing NAFLD; unlike they found no association between TCF7L2rs7903146 Gene Polymorphism and NAFLD, CAD, and NAFLD + CAD in their study.

Due to the close association of NAFLD with abdominal obesity, DM, HT, dyslipidemia, and insulin resistance, some researchers recommend to change the definition of this disease to metabolic associated fatty liver disease (MAFLD). Herbert T et al.^28^ reported a new article in 2020 and they defending that two new position papers convincingly propose that NAFLD needs a new name MAFLD. They reported that a new name for this disease affecting nearly one billion people globally is overdue, as knowledge gained from the past decades has assuringly demonstrated that MAFLD is a purely metabolic disorder. NAFLD reflects a progressive condition in many instances and its prevalence parallels trends in obesity and diabetes. Eslam M at al.^29^ writed a letter to the editor in 2020 for thehange of the name NAFLD and claimed that MAFLD definition provides a meaningful working definition and conceptual framework for approaching the disease that is consistent with our evolving understanding of its pathophysiology. Lonardo A. et al.^30^ published another systematic review of the literatüre about history of NAFLD in 2020 and reported NAFLD and MAFLD are not exactly the same disease and they reported that MAFLD is more likely to capture those patients with hepatic steatosis, who exhibit a higher risk of disease progression. Although there are a few articles in the literature advocating the change of name as MAFLD, it was observed that there is still no definite consensus on this issue.

Aspartate aminotransferase is a cytoplasmic enzyme that catalyzes the transfer of the amino group of aspartic acid to ketoglutaric acid. This enzyme is found most abundantly in myocardial cells second to hepatic cells.^31^ Hence, serum AST level rises early after injury to these tissues. The increase in AST level is proportional to the extent of cellular injury and thus it is an important serum parameter for monitoring of injury severity or improvement. A significant rise occurs in serum AST level within 6–8 hours following MI and reaches its peak by 48–60 hours. In our study, some patients were admitted for ACS who were expected to have elevated AST level.^32^ Masoudkabir et al.^33^ found a relationship between AST, ALT levels and angiographically proven CAD and CAD extent and severity assessed by Gensini score. We found a significant relationship between AST level and both angiographically proven CAD and Gensini-indicated CAD extent and severity. Many risk factors for CVD are reportedly associated with serum GGT elevation.^34^ Shabbir et al.^35^ found a significantly increased GGT activity in patients with CAD and reported a positive correlation between GGT level and blood pressure, serum glucose and cholesterol level, and smoking. Similarly, Aksakal et al.^36^ reported that serum GGT level was correlated to coronary lesion complexity and long-term mortality among patients with stable CAD. In our study a correlation analysis between Gensini score and GGT level found a p value of borderline significance (p= 0.051).

NAFLD is detected upon demonstration of hepatic fatty infiltration when a history of alcohol abuse or other secondary chronic liver disease is absent. We also excluded any patient with a history of alcohol or a secondary cause. Whereas today liver biopsy is considered gold standard to make the diagnosis of hepatosteatosis, we made use of abdominal ultrasonography, which is a noninvasive and quickly performed method and thus the most commonly ordered imaging study to diagnose hepatosteatosis.^37^ Joy et al.^38^ reported that USG had a good sensitivity (89%) and specificity (93%) for determining moderate-to severe hepatosteatosis. Our study also enrolled patients in whom the presence of NAFLD was investigated with USG.

## Conclusion

The existence and severity of ultrasonographically detected hepatosteatosis may independently affect both the presence and severity of CAD. Nevertheless, obesity may not have been linked to the presence and severity of CAD. Finally, serum AST and GGT increase may be an independent indicator of CAD.

## Limitations of the study

Firstly, we did not histologically confirm hepatosteatosis. We are unable to clearly establish any link between histological findings and metabolic abnormalities. Although abdominal ultrasonography is fairly sensitive and specific for moderate-to-severe hepatosteatosis, it has a lower sensitivity when biopsy indicates that hepatic fatty involvement is below 33%.^39^ Hence, we may have made conservative estimates of any association between NAFLD and CAD. Secondly, as for the clinical properties of the patients, they were examined with coronary angiography at our hospital for different reasons, one of which is ACS. Hence, we cannot establish any association between patients with and without ACS. For instance, AST and CAD may not be independently associated in patients not suffering an MI. AST and the other variables may need to be studied separately in ACS and non-ACS settings.
